# Socioeconomic Status and Access to Care for Pediatric and Adult Congenital Heart Disease in Universal Health Coverage Models

**DOI:** 10.3390/jcdd11080250

**Published:** 2024-08-16

**Authors:** Amanda A. Greenwell, Mimi X. Deng, Shelagh Ross, Viktoria Weixler, Dominique Vervoort

**Affiliations:** 1 Temerty Faculty of Medicine, University of Toronto, Toronto, ON M5S 1A8, Canada; 2 Division of Cardiac Surgery, University of Toronto, Toronto, ON M5S 1A1, Canada; 3 Division of Cardiovascular Surgery, The Hospital for Sick Children, Toronto, ON M5G 1X8, Canada; 4 Global Alliance for Rheumatic and Congenital Hearts, Victoria, BC V8S 4N9, Canada; 5 Institute of Health Policy, Management and Evaluation, University of Toronto, 155 College St 4th Floor, Toronto, ON M5T 3M6, Canada

**Keywords:** congenital heart disease, congenital heart surgery, health equity

## Abstract

Congenital heart disease (CHD) is the most common major congenital anomaly, affecting one in every 100 live births. Whereas over 90% of children born with CHD in low- and middle-income countries cannot access the care they need, early detection, advances in management, and financial risk protection have resulted in over 90% of children with CHD in high-income countries surviving into adulthood. Despite the presence of universal health coverage, barriers to accessing high-quality cardiovascular and non-cardiovascular care for CHD remain common. Lower socioeconomic status has been associated with differential access to cardiac care and poorer outcomes across multiple cardiovascular conditions and subspecialties. In this review article, we describe the relationship between socioeconomic status and access to CHD care in countries with universal health coverage models. We further evaluate notable challenges and opportunities to improve equitable, high-quality CHD care in these countries.

## 1. Background

Congenital heart disease (CHD) affects approximately one in every 100 live births and represents a heterogeneous group of conditions with a range of medical and surgical needs [1,2]. An estimated one-third of CHD are critical CHD, requiring life-saving care within the first year of life, whereas approximately 49% of individuals with CHD will require surgery or interventional cardiology at least once in their lifetime [3]. Despite over one million children born with CHD in low- and middle-income countries every year, more than 90% are unable to access the care they need [2,4]. Although CHD forms an increasingly leading cause of mortality among neonates and children in low- and middle-income countries, over 95% of those born with CHD in high-income countries (HIC) now survive into adulthood [2]. However, gaps in care for CHD are not absent in HICs. In the United States, disparities in access and the financial burdens of healthcare result in variable timing of health-seeking behaviors and additional physical and mental health-related challenges [5,6]. In other HICs, namely those with universal health coverage (UHC) models, gaps present differently but are poorly studied [7,8]. Socioeconomic status (SES) is one key sociodemographic factor associated with cardiovascular burdens, care, and outcomes, requiring further study and action in the context of CHD [9].

In this narrative review, we describe the burden of congenital heart disease with a focus on HICs with UHC models. We summarize the current evidence on access to and disparities in CHD care across a lifetime in these contexts and conclude by highlighting major challenges and opportunities to work towards leaving no patient behind.

## 2. Global Burden of CHD/ACHD

Approximately 12.0 million people live with CHD worldwide, with an incidence of 1.35 per year [10]. In HICs, the burden of CHD is concentrated in adults, whereas in low- and middle-income countries, children bear the brunt of the burden. Table 1 summarizes the burden of CHD by sex and age among HICs with UHC models [11]. Health systems with UHC models enable reduced barriers to and delays in seeking care, resulting in earlier and higher rates of detection of CHD and the ensuing ability to timelier intervene when needed. As a result, adult CHD (ACHD) is becoming widely prevalent, with the prevalence of ACHD now surpassing CHD in children in HICs. Despite this surge and the unique needs that are associated with ACHD, there remains insufficient ACHD capacity globally, including in HICs [12]. At the same time, improvement in outcomes of childhood CHD should not undermine the impact of SES on CHD prevalence or access to care, which undoubtedly has implications on patients’ health status in adulthood.

In a Californian population-based study, social deprivation and environmental exposures to pollutants correlated with CHD prevalence, with an odds ratio (OR) of 1.31 [95% confidence interval: 1.21–1.41] and 1.23 [1.15–1.31], respectively [13]. Countries with UHC demonstrate similar trends. In a United Kingdom study of 5350 infants who underwent surgical repair for CHD, disease incidence was significantly higher in infants of Asian and Black ethnicities, compared with the Caucasian reference population [14]. The CHD incidence risk ratio of CHD was 1.5 [1.4–1.7] for Asian and 1.4 [1.3–1.6] for Black patients. Although rates of antenatal diagnosis and age at intervention were comparable between ethnic groups, affected children from non-White ethnic groups were more likely to live in addresses associated with low SES, for which over 50% of Asian and Black patients belonged to the most-deprived quantile [14]. A study by Miao et al. of 9359 CHD infants born in Ontario, Canada, found that mothers in the quintile of highest material deprivation had an adjusted OR of 1.27 [1.18–1.37] for having an infant with CHD, compared to the quintile with lowest material deprivation [15,16]. This supports previous reports of *d-*transposition of the great arteries being associated with food insecurity [17]. Interestingly, ethnic concentration, defined as the geographic density of people who identify as an immigrant and/or part of a visible minority group, was inversely related to CHD incidence [15,16]. These findings build on the authors previous work signaling increased odds of CHD in settings of low maternal neighborhood household income, poverty, educational level, and unemployment status [18]. In an Ontario-based study from 1994 to 2007, low SES, defined by a composite of income and maternal education level, was strongly associated with CHD (relative risk 1.20 [1.15–1.24]) [15,16]. The prevalence of CHD decreased over time, with the exception of non-severe CHD in low-SES families. A more recent Canadian ecological study continues to report an inverse relationship between major CHD diagnoses and SES quintiles, specifically for CHD phenotypes of double-outlet right ventricle, hypoplastic left heart syndrome, heterotaxy, tetralogy of Fallot, truncus arteriosus, and ventricular septal defect [19]. In one of the largest national cohort studies on CHD and SES involving nearly 750,000 patients, 1.8 and 2.2 per 1000 Swedish children hospitalized for CHD were from the least and most deprived neighborhoods, respectively [20]. CHD hospitalization positively correlated with neighborhood-level deprivation for all family- and individual-level sociodemographic groups with an OR of 1.23 [1.04–1.46]. Across healthcare systems, low SES remains a prominent risk factor for the development of CHD.

## 3. Defining Access to Care

**Figure 1:**
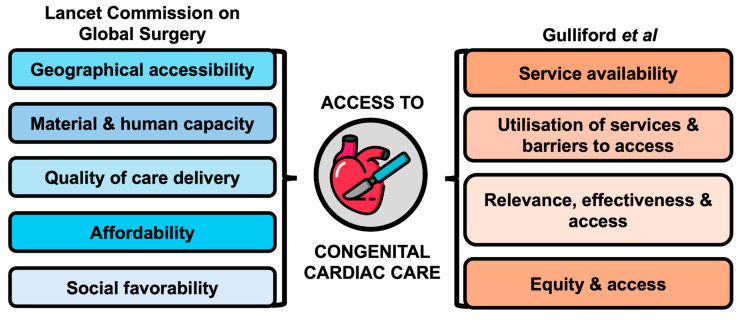
Access to high-quality congenital cardiac care [23].

A key component of studying access to care is defining the concept of access. Despite its semantic focus on physical access, true access is a function of individual but indispensable components. The Lancet Commission on Global Surgery described access to care as ensuring geographical accessibility, resource (including workforce) availability, care quality, and financial affordability [21]. This may be extended to include a fifth pillar, being the social acceptability of care within a community [22]. Access to care can further be approached through the framework by Gulliford et al. [23], focusing on service availability, service utilization, relevance and effectiveness, and equity. For the purposes of this review, both frameworks are merged to holistically study access to CHD care (Figure 1).

**Figure 2:**
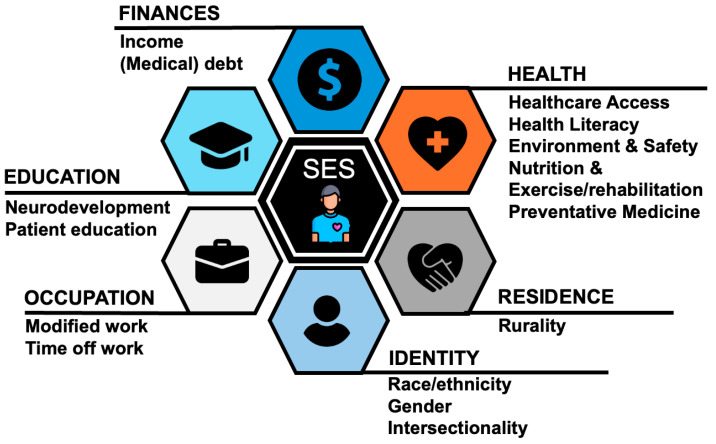
Effects of sociodemographic and socioeconomic status (SES) on access to care for individuals living with congenital heart disease.

Cardiovascular diseases, in particular CHD, are unique conditions that can be treated but generally cannot be cured. As such, current professional guidelines recognize the need for lifelong care and access to specialized care for individuals living with CHD, assigning it the highest possible recommendation (Class I) [24]. While variations may occur, it can be assumed that care is largely accessible (with exceptions of parts of larger countries and island states), well-resourced, high-quality, financially affordable (due to UHC), and socially accepted in HICs. However, utilization, and especially equity, may be limited due to various structural and systemic barriers, as well as sociodemographic factors. Herein, the role of SESs, both in isolation and in association with other sociodemographic factors (e.g., rurality, race/ethnicity), must be recognized (Figure 2). These include preventive and health-promoting factors such as the (in)ability to purchase healthy foods and engage in physical activity, exposure to negative environmental factors and a lack of green surroundings, and health literacy, among others. Importantly, these also include social safety nets, enabling people to take time off work, visit healthcare workers and facilities, and have the support to pursue health-promoting behaviors.

## 4. Socioeconomic Status and Disease Detection

Prenatal diagnosis serves an important role in reducing morbidity and mortality related to CHD [25]. Across countries with UHC models, including France, Canada, Slovakia, Italy, and the United Kingdom, prenatal detection rates ranged from 21% to 88% [26]. Variability in rates may be attributed to differences in prenatal ultrasound screening protocols and consequent referral rates for fetal echocardiography [26,27]. However, several lines of evidence also point towards sociodemographic factors, including SES, as significant barriers contributing to suboptimal prenatal CHD detection rates. A Canadian study found that in the metropolitan setting, the lowest neighborhood-level SES quintile was associated with a 24% and 46–66% higher rate of missed prenatal diagnosis and late prenatal diagnosis (≥22 weeks), respectively, when compared to the highest SES quintiles [28]. Although rural residence (≥100 km from a tertiary fetal cardiology center) was associated with a higher rate of missed and late prenatal diagnosis, these parameters were not further elevated by SES disparities among rural residents. Another study of the two largest centralized fetal cardiology and surgical centers in Canada additionally demonstrated an association between lower SES and late gestational age at prenatal diagnosis of hypoplastic left heart syndrome and transposition of the great arteries [29]. However, there was no evidence of ethnic variation in the prenatal diagnosis rates of CHD in England and Wales, despite non-White patients being more likely to reside within areas representing the highest socioeconomic deprivation [14]. Financial barriers related to childcare, transportation, inflexible work schedules, and lower wages may limit access to prenatal screening for patients with lower SES. The cost of travel to specialized healthcare centers from rural communities may further compound the financial burden.

## 5. Socioeconomic Status and Access to Care

For systems with a UHC model, despite a foundational focus on equitable access to health services, ensuring timely and adequate healthcare access still remains a challenge. Although publicly funded healthcare systems can alleviate some SES-related barriers to accessing optimal CHD care, several lines of evidence point to an association between SES (and other related sociodemographic factors) and disparities in CHD outcomes.

A study by Olugbuyi et al. reported that the risk of mortality was highest among infants hospitalized for moderate CHD who resided in the most remote (≥300 km from the closest cardiac surgical center) and socioeconomically deprived areas within Canada [30]. Conversely, among infants with severe CHD, hospital mortality was not found to be associated with SES or remoteness of residence. These findings suggest that UHC in Canada is more effective in mitigating sociodemographic barriers to care for infants from vulnerable backgrounds with severe, rather than moderate complexity, CHD. Delayed diagnosis attributed to missed prenatal detection for moderate CHD subtypes that are challenging to detect with obstetrical ultrasound, and referral and scheduling delays for surveillance and interventions for lower acuity cases may underlie the differences in findings between moderate and severe CHD. These factors are likely accentuated for families of lower SES residing in remote communities where prenatal and postnatal cardiac care are less accessible [31]. Whether these findings can be extrapolated to other UHC systems requires further investigation. In England and Wales, no significant association between mortality and SES was reported for infants with hypoplastic left heart syndrome; however, this study did not compare these results with other CHD subtypes [32].

Children and adults with CHD are also at an increased risk for having extracardiac comorbidities, including neurodevelopmental impairments and psychiatric disorders, the outcomes of which can be impacted by SES. Neurocognitive assessments performed in children aged 48–72 months undergoing surgical palliation for hypoplastic left heart syndrome in Alberta, Canada, reported that low SES was associated with lower performance intelligence quotient scores [33]. Furthermore, low and high parental socioeconomic and educational status predicted poorer neuropsychological performance and increased lifetime prevalence of anxiety disorders, respectively, among a population of French adults who received arterial switch operation for *d-*transposition of the great arteries as a child [34].

Mitigating the impacts of SES on access to care for patients with CHD is challenging given the broad definition of SES and intricate association with other sociodemographic factors. Establishment of CHD outreach clinics, use of telemedicine, and creation of CHD-specific educational programs for local primary care providers represent a sampling of strategies that could be employed to improve the accessibility of cardiac screening and care in remote communities. Partnerships between CHD-trained cardiologists and community health programs to improve surveillance and support of patients with CHD, as well as the formation of holistic healthcare teams to address and monitor for CHD-related comorbidities, may assist in addressing the unique needs of patients with CHD and ensure equitable healthcare access.

## 6. Socioeconomic Status and Lifelong Care

With innovations in imaging, medical therapies, and surgical techniques, almost 90% of children diagnosed with CHD now survive into adulthood [35]. As the prevalence of ACHD continues to rise, so does the critical need for longitudinal and specialized ACHD care in order to monitor and manage the lifelong cardiac and extracardiac disease burden in this population. Indeed, cardiology follow-up in patients with ACHD improved survival rates and decreased the risk of major cardiac events when compared to follow-up with primary care providers only [36]. Expectedly, many patients experience a lapse in care or a complete loss to follow-up, especially during the vulnerable transition period from pediatric to adult cardiac care [37,38,39,40]. A recent meta-analysis estimated the proportion of discontinuity of care among young individuals with CHD to be nearly 26% in Canada [37,38,39]. In contrast, only 7.3% of patients with ACHD were lost to cardiac follow-up in Belgium [41]. There exists a lack of healthcare infrastructure in more remote Canadian and often Indigenous communities. Variable internet access limiting telehealth expansion, decentralized electronic health record systems, long waitlist times, and fragmentation and turnover of provincial healthcare governance disadvantage remote communities from accessing timely care [8]. Geographic differences between Canada and Belgium may partially account for this discrepancy and highlight the impact of sociodemographic factors, including remoteness of residence, on the transition from pediatric to adult cardiac care. Beyond geography, low SES represents a notable barrier to a successful transition of care. Higher family income was identified as a protective variable from loss to follow-up in a Canadian study of children and adults with CHD (OR 0.87 per $10,000 increase [0.77–0.98]) [42].

Beyond ancillary out-of-pocket healthcare costs that can disproportionately affect lower-income families and contribute to missed and delayed follow-up appointments, socioeconomic barriers may impact transition readiness in adolescents and young adults. Among patients with CHD aged 12–15 years, increased patient knowledge of their CHD was correlated with increased transition readiness based on a self-assessment questionnaire [38]. Greater parental involvement in childhood can improve patient health literacy and disease management skills, thus affording patients the critical knowledge, resources, and skills to manage their unique healthcare needs and navigate the complexities of the healthcare system as an adult. Although household income was not significantly correlated with patients’ transition readiness scores in the aforementioned study or the successful transfer to adult care in another Canadian study [43], SES may inevitably impact the level of parental involvement and access to educational resources, which can influence transition readiness in some patients.

Socioeconomic disparities within UHC systems can additionally affect the capability of individuals with CHD to thrive in educational environments. In the United Kingdom, educational attainment was poorer in children with CHD compared with age-matched children with no congenital abnormalities and even lower in children with CHD whose parents were receiving social benefits [44]. Children with CHD from less affluent socioeconomic backgrounds were also less likely to access special educational needs support. Studies from France [45] and Finland [46] provide evidence that impaired academic achievement in early childhood among individuals with CHD can manifest as reduced rates of higher education and employment in early adulthood, thus further accentuating socioeconomic barriers to accessing lifelong ACHD care.

Even within UHC systems, SES may present barriers to the continuity of care for patients with CHD. Addressing these disparities requires dedicated patient education and transition programs with integrated social services to prevent loss-to-follow-up and better support for patients’ health, educational, and social needs across the lifespan.

## 7. Challenges and Opportunities

A common challenge in assessing the impact of SES on health outcomes is the lack of consensus for its definition. SES is the amalgamated measure of an individual’s position within the economy and society at large, with both these components being umbrella terms that can be further deconstructed. Educational level, income, and occupation are the three foundational components of SES, with or without other ancillary criteria [47]. SES is closely associated with other social determinants of health (including race/ethnicity, sex/gender, geography, Indigeneity, etc.), such that demarcating the precise boundaries of SES is often impractical [34]. Nonetheless, variations in the definition of SES make it difficult to interpret evidence across different countries, regions, and even institutions. Additionally, using SES to infer poverty and marginalization can be overly reductive. Although pragmatic, the absence of a standardized definition for SES limits the reproducibility of results and the validity of conclusions.

The multifaceted nature of SES also means that there is no panacea or silver bullet to removing systemic barriers to CHD/ACHD care. Low income, environmental exposures, food deserts, and lack of household support are not easily modifiable risk factors. Tackling the root of socioeconomic shortcomings demands a multidisciplinary and multipronged approach that extends beyond hospital walls. In people with CHD where the burden of disease can be lifelong, it is also important to recognize that the social determinants of health need to be addressed early to avoid impairment of childhood development, which is closely tied to functional independence as an adult [48]. Beyond patient-level factors, the patient’s family is also an integral part of receiving timely and effective care, especially for pediatric patients. Education and counseling should routinely involve the family unit when appropriate. By extension, engagement of patient advocates and patient–family organizations, such as the Global Alliance for Rheumatic and Congenital Hearts (Global ARCH) and its member organizations globally, can further promote patient-centered care delivery [49]. However, even within patient organizations, barriers that arise from SES persist. Although many patient organizations in HICs have become more diverse over years due to concerted efforts for more inclusive representation, there remains a lack of participation by racialized and Indigenous individuals, and the leadership is often still dominated by more affluent and educated members [50]. Moreover, there is a paucity of patient-friendly educational information adapted for individuals with low health literacy [49]. Finally, participation in patient organizations and most philanthropic activities requires leisure time and financial means, as well as possibly childcare, computer/internet access, facilities, social capital, and awareness of the resources available. As such, efforts to understand and address the impact of SES should not preclude the very non-profit organizations that advocate for equitable provision of care.

Financial investment in research and timely implementation of new, cutting-edge treatments and technologies remain key areas for improvement, more specifically in UHC countries compared to market-based health systems (e.g., the United States), where private industry and competition in healthcare more directly spur innovation. Furthermore, in comparison to non-UHC countries, decentralized health services, as is present in the United States, may offer expanded provider choice and geographic access compared to large UHC countries such as Canada. However, an increase in lower-volume surgical centers can adversely affect surgical outcomes due to the established relationship between increased hospital surgical volume and improved outcomes [51]. In addition, decentralization can further contribute to the growing SES divide, as evidence suggests that many patients will still choose to accept the travel burden of seeking treatment at high-volume surgical centers rather than at their closest hospital, likely due to the volume–outcome association [52].

**Figure 3:**
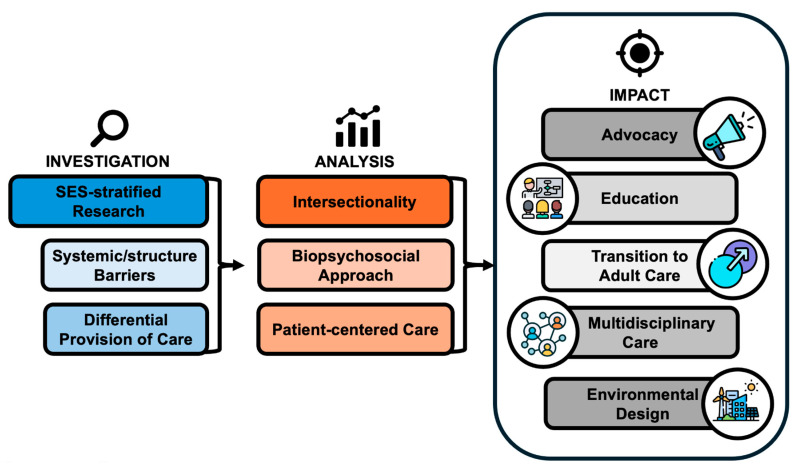
Opportunities to improve cardiovascular and non-cardiovascular care for individuals living with congenital heart disease and lower socioeconomic status. SES, socioeconomic status.

Stratified analysis can provide a starting point for understanding complex problems with many moving parts, such as the impact of SES on CHD/ACHD management (Figure 3). SES subgroup analysis for health outcomes research and distributional cost-effectiveness analyses can identify systemic barriers and inform policy change. Furthermore, understanding trends in referrals, surgical techniques, and postoperative follow-up can help identify areas of clinical equipoise and advise guideline development. Once issues on the system levels are characterized, it is important to redirect the focus back to the patient and apply shared decision-making when constructing treatment plans. Furthermore, proper patient-centered care requires an intersectionality lens that takes into consideration the totality of the patient identities, experiences, and traditions to proactively evaluate the challenges patients might encounter while navigating the healthcare system [35]. This includes taking measures to ensure that the vulnerable period in transitioning from pediatric to adult cardiac care is seamless and retains access to specialty services. CHD in childhood can directly impact a patient’s future wellbeing through the need for reintervention or indirectly through developmental delays due to a protracted hospital stay during a patient’s formative years. The more elusive biopsychosocial sequelae of pediatric CHD affect not just the patients but also their families, and caregiver burden should not be overlooked [53]. The numerous stakeholders and multisystem involvement of this special patient group calls for an interdisciplinary approach to care that encompasses non-cardiovascular health services for mental health, neurodevelopmental, and social work needs [37,38]. Widening this purview even further, the environments that support patients after discharge from hospital underpin and sustain their health status. Urban planning of green spaces and government programs for health-promoting behaviors (e.g., subsidies for gym memberships, digital health technologies, hospital transportation, health literacy campaigns) are ways to improve a population’s collective health. There is no singular solution to optimizing the socioeconomic health of patients with CHD/ACHD, but concerted and diversified efforts in multiple spheres can make a difference that is greater than the sum of its parts.

## 8. Conclusions

The prevalence of CHD and ACHD is growing, and the disease burden throughout a patient’s lifetime should not be underestimated. Beyond the surgical management itself, CHD/ACHD is unique because of its pervasive impact on the patient’s biopsychosocial development and caregiver relationships, as well as the intensity and duration of healthcare utilization. In UHC settings, SES remains a critical but poorly modifiable risk factor for the embryological development of CHD and affects a patient’s trajectory at the level of diagnosis, treatment, and postoperative surveillance. Similarly, in countries without UHC, structural efforts should not only focus on expanding healthcare coverage in itself but simultaneously recognize the effects of SES throughout a lifetime. Addressing health disparities associated with SES requires active reduction in systemic barriers and mobilization of multidisciplinary care teams to ensure quality of care and follow-up.

## Figures and Tables

**Table 1:** Prevalence, incidence, death, and disability-adjusted life-year (DALY) rates from congenital heart disease in high-income countries with universal healthcare coverage, stratified by country, sex, and age. Values indicate cases per 100,000 population. Note: Countries were selected as examples of Beveridge (e.g., United Kingdom, Sweden), Bismarck (e.g., Germany, Japan), and national health insurance (e.g., Canada, South Korea) models.

	Population	Prevalence	Incidence	Death	DALYs
Canada	Overall	136.0	7.4	0.7	56.4
	Male	126.3	6.8	0.8	66.0
	Female	145.4	7.9	0.5	47.2
	<20 y.o.	298.8	33.7	1.6	175.6
	>20 y.o.	90.2	-	0.4	22.9
South Korea	Overall	219.4	6.2	0.3	33.6
	Male	203.5	6.0	0.3	33.3
	Female	235.4	6.4	0.3	33.9
	<20 y.o.	339.9	38.0	1.0	118.8
	>20 y.o.	196.0	-	0.2	17.0
United Kingdom	Overall	213.2	10.5	0.7	62.3
	Male	204.6	11.1	0.8	71.9
	Female	221.4	9.9	0.6	53.0
	<20 y.o.	322.8	45.4	1.8	182.5
	>20 y.o.	180.3	-	0.4	26.2
Japan	Overall	295.4	9.5	0.4	44.4
	Male	290.6	9.7	0.5	50.0
	Female	299.9	9.4	0.4	39.1
	<20 y.o.	417.9	57.4	1.3	148.8
	>20 y.o.	270.9	-	0.3	23.6
Germany	Overall	285.3	11.8	0.6	55.5
	Male	286.3	12.6	0.6	61.2
	Female	284.3	11.0	0.5	49.8
	<20 y.o.	434.3	63.0	1.8	198.1
	>20 y.o.	251.1	-	0.3	22.7
Australia	Overall	159.3	10.3	0.6	50.8
	Male	152.6	10.6	0.7	59.3
	Female	165.9	10.1	0.5	42.4
	<20 y.o.	291.6	42.8	1.3	143.2
	>20 y.o.	117.1	-	0.3	21.3

## Abbreviations

ACHD	Adult congenital heart disease
CHD	Congenital heart disease
HIC	High-income country
OR	Odds ratio
SES	Socioeconomic status
UHC	Universal health coverage
